# Low micromolar Ba^2+^ potentiates glutamate transporter current in hippocampal astrocytes

**DOI:** 10.3389/fncel.2013.00135

**Published:** 2013-08-28

**Authors:** Ramil Afzalov, Evgeny Pryazhnikov, Pei-Yu Shih, Elena Kondratskaya, Svetlana Zobova, Sakari Leino, Outi Salminen, Leonard Khiroug, Alexey Semyanov

**Affiliations:** ^1^Neuroscience Center, University of HelsinkiHelsinki, Finland; ^2^Faculty of Pharmacy, Division of Pharmacology and Toxicology, University of HelsinkiHelsinki, Finland; ^3^RIKEN Brain Science InstituteWako-shi, Japan; ^4^Department of Neurodynamics and Neurobiology, University of Nizhny NovgorodNizhny Novgorod, Russia

**Keywords:** glutamate transporters, barium, glutamate uptake, astrocytes, hippocampus

## Abstract

Glutamate uptake, mediated by electrogenic glutamate transporters largely localized in astrocytes, is responsible for the clearance of glutamate released during excitatory synaptic transmission. Glutamate uptake also determines the availability of glutamate for extrasynaptic glutamate receptors. The efficiency of glutamate uptake is commonly estimated from the amplitude of transporter current recorded in astrocytes. We recorded currents in voltage-clamped hippocampal CA1 *stratum radiatum* astrocytes in rat hippocampal slices induced by electrical stimulation of the Schaffer collaterals. A Ba^2+^-sensitive K^+^ current mediated by inward rectifying potassium channels (K_ir_) accompanied the transporter current. Surprisingly, Ba^2+^ not only suppressed the K^+^ current and changed holding current (presumably, mediated by K_ir_) but also increased the transporter current at lower concentrations. However, Ba^2+^ did not significantly increase the uptake of aspartate in cultured astrocytes, suggesting that increase in the amplitude of the transporter current does not always reflect changes in glutamate uptake.

## Introduction

Glutamate is the major excitatory neurotransmitter in the brain. After synaptic release, this neurotransmitter is quickly cleared by diffusion and uptake (Bergles and Jahr, 1998). Uptake is mediated by glutamate transporters powered by the transmembrane K^+^ and Na^+^ gradients. Glutamate transporters belong to several groups (EAAT1-5) and are expressed in both neurons and astrocytes. In addition to uptake, these transporters are involved in buffering (Diamond and Jahr, 1997) and release of glutamate in a process termed “reversed uptake” (Rossi et al., 2000; Grewer et al., 2008). All of these processes shape the glutamate concentration profile in the synaptic cleft, regulate activation of high-affinity extrasynaptic glutamate receptors, and play a crucial role in spillover and intersynaptic crosstalk (Kullmann and Asztely, 1998; Bergles et al., 1999; Diamond, 2001; Zheng et al., 2008). Efficient glutamate transporters also separate the synaptic and extrasynaptic signaling pathways by “shielding” synapses from extrasynaptic glutamate (Lozovaya et al., 2004; Wu et al., 2012). Failure to remove glutamate after synaptic release, as well as activation of reversed uptake (e.g., under ischemic conditions) can lead to glutamate accumulation in the extracellular space, leading to uncontrolled excitation, epileptiform discharges, and eventually neuronal death (glutamate cytotoxicity), which is linked to severe neurodegenerative diseases (During and Spencer, 1993; Schousboe and Waagepetersen, 2005).

Although both neurons and astrocytes express glutamate transporters, the uptake capacity of astrocytes is much higher than that of neurons (Rothstein et al., 1996; Danbolt, 2001). Deficiencies of astrocytic glutamate uptake are directly implicated in the pathogenesis of several neurologic disorders, such as amyotrophic lateral sclerosis (Rothstein et al., 1992), Alzheimer's disease (Masliah et al., 1996), Parkinson's disease (Blandini et al., 1996), and epilepsy (Meldrum, 1994). Reduced transporter activity is also reported in the hippocampi of patients with pharmaco-resistant epilepsy, which comprise up to 1/3 of all epilepsy patients (Regesta and Tanganelli, 1999; Proper et al., 2002).

Because glutamate transport is electrogenic, the efficiency of astrocytic glutamate transporters is commonly estimated by measuring the transporter current evoked in voltage-clamped astrocytes by synaptic stimulation (Bergles and Jahr, 1997; Diamond and Jahr, 2000; Tanaka et al., 2013). Another major component of astrocytic current is mediated by inward rectifying potassium channels (K_ir_) (Kofuji and Newman, 2004). This component is sensitive to Ba^2+^. Here, we found that Ba^2+^ also increases the amplitude of glutamate transporter current but does not affect aspartate uptake by these transporters in hippocampal astrocytes.

## Methods

### Preparation and maintenance of slices

Sprague Dawley rats (postnatal days 21–28) were anesthetized with halothane prior to decapitation. Hippocampi were dissected and 400-μm transverse hippocampal slices were cut using a VT 3000 vibratome (Leica, Wetzlar, Germany) in ice-cold cutting solution containing (in mM): 50 sucrose, 87 NaCl, 3 KCl, 0.5 CaCl_2_, 25 NaHCO_3_, 1 NaH_2_PO_4_, 7 MgCl_2_, and 25 D-glucose. Slices were allowed to recover at 36°C for 1 h in storage solution containing (in mM): 124 NaCl, 3 KCl, 1 CaCl_2_, 25 NaHCO_3_, 1 NaH_2_PO_4_, 3 MgCl_2_, and 25 D-glucose (gassed with 95% O_2_ and 5% CO_2_, pH 7.4). One slice was then placed in a submerged-type recording chamber (volume, ~ 2 ml) and continuously perfused at 2 ml/min and at 34°C during the experiments with normal physiologic solution containing (in mM): 124 NaCl, 3 KCl, 2 CaCl_2_, 25 NaHCO_3_, 2 MgCl_2_, and 10 D-glucose.

### Electrophysiology

Whole-cell patch-clamp recordings were performed in visualized astrocytes using an EPC 10 amplifier (HEKA Elektronik, Germany) or Multiclamp 700B amplifier (Molecular Devices, USA). The patch pipette solution contained (in mM): 125 K-gluconate, 19 KCl, 10 NaCl, 2 Mg-ATP, 0.3 EGTA, 0.5 Na-GTP, 10 phosphocreatine, and 10 HEPES, and the pH was adjusted to 7.3 with KOH. Patch pipettes were fabricated from borosilicate glass (Harvard Apparatus, UK), with resistance ranging from 6 to 8 MΩ. For recordings of synaptically-induced astrocytic current, 50 μ M DL-2-amino-5-phosphonovalerate (DL-APV) and 10 μ M 6-cyano-7-nitroquinoxaline-2,3-dione (CNQX; Sigma-Aldrich, USA) were added to the solution. In experiments with caged MNI-glutamate, 0.5 μ M tetrodotoxin (Tocris Bioscience, USA) was also added. In one set of experiments, we used a Cs-based intracellular solution containing (in mM): 125 Cs-gluconate, 19 CsCl, 2 Mg-ATP, 5 BAPTA, 0.5 Na-GTP, and 10 HEPES (pH adjusted to 7.3 with CsOH).

### Local photolysis of caged glutamate

Glutamate was locally photolyzed from the caged MNI-glutamate (Sigma-Aldrich) as described previously (Khirug et al., 2005). Caged MNI-glutamate (2 mM) was dissolved in the physiologic solution and delivered at a flow rate of 1 μ l/min to the vicinity of the patch-clamped cell using an UltraMicroPump II syringe pump (WPI, USA) and a syringe tip with an inner diameter of 100 μm. Local photolysis of caged MNI-glutamate was performed with laser light (375 nm, Oxxius, France) through an Olympus LUMPlanFl 60× water-immersion objective. The beam yielded an uncaging spot of ~10 μm in diameter that was focused at the soma. The laser power (1–5 mW at the objective output) and flash duration (10–20 ms) were set at a level that provided a good signal-to-noise ratio for uncaging-evoked currents. Control experiments showed that the laser flash evoked no responses in the absence of the caged glutamate.

### Astrocyte culture

Primary cortical astrocyte cultures were prepared from the cerebral cortex of P2 Wistar rats. Cells were dissociated with mechanical trituration and papain in a Ca^2+^- and Mg^2+^-free balanced salt solution (HBSS, pH 7.4), supplemented with 1 mM sodium pyruvate and HEPES. After centrifugation at 1000 rpm for 5 min, the cells were suspended in high-glucose Dulbecco's modified Eagle's medium (DMEM; Lonza, Switzerland) supplemented with 10% fetal bovine serum and 25 μg/mL penicillin/streptomycin. Astrocytes were plated at a density 30 × 10^3^ cells/cm^2^ on 6-well Corning Costar cell culture plates, pre-treated with poly-L-lysine (1–2 μg/cm^2^). Cells were grown in 5% CO_2_/95% air atmosphere at 37°C. The day of plating was designated as day-*in-vitro* 0 (DIV0). Medium was changed every 3–4 days.

### Uptake assay in cultured astrocytes

A modification of a previously described method (Kimelberg et al., 1990) was used to investigate aspartate uptake in cultured astrocytes. All steps were performed at room temperature, unless otherwise specified. Astrocytes (23–25 days *in vitro*) growing on 6-well plates were washed once with warm uptake buffer and then pre-incubated in 1 ml/well of uptake buffer for 15 min at 37°C and 5% CO_2_. The uptake buffer contained (in mM): 135 NaCl, 5 KCl, 2.5 CaCl_2_, 0.6 MgCl_2_, 6 D-glucose, and 10 HEPES, and the pH was adjusted to 7.40 with NaOH. Subsequently, uptake was initiated by replacing the pre-incubation buffer with 1 ml/well of uptake buffer containing the substrate [20 μM D-aspartic acid (Sigma-Aldrich), 1% of which was D-[2,3-^3^H]-aspartic acid (PerkinElmer; 10.0 Ci/mmol)] either alone or in combination with 1 μM of BaCl_2_ (Sigma-Aldrich) or 2 μM of the EAAT_1/2_ inhibitor (3S)-[[3-[[4-(trifluoromethyl)benzoyl]amino]phenyl]methoxy]-L-aspartic acid (TFB-TBOA; Tocris Bioscience). Uptake was continued for 20 min at 37°C and 5% CO_2_ and then terminated by placing the cells on ice and washing rapidly three times with ice-cold 0.32 M sucrose. The cells were then immediately solubilized in 0.25 M NaOH (1 ml/well). The amount of aspartate retained by the cells was determined by measuring the radioactivity of the solubilized samples by scintillation counting using a Wallac WinSpectral 1414 liquid scintillation counter after adding Optiphase HiSafe 3 scintillation cocktail (PerkinElmer). Instrument efficiency was 40%. Protein concentration in the samples was measured with the Bradford assay using the Sigma-Aldrich Bradford Reagent. Data are expressed in picomoles of substrate per milligram of protein (pmol/mg).

### Plasmids

CMV-hEAAT_1_ and CMV-hEAAT_2_ plasmids were prepared in Susan Amara lab and ordered from Addgene (non-profit organization for sharing of plasmids, Cambridge, MA, USA; see Addgene plasmids 32813 and 32814, respectively, for detailed information and sequences).

### HeLa cell culture and transient transfection

Cell culture plasticware was obtained from Nunc. Culture medium was purchased from Sigma-Aldrich and media supplements were obtained from *Invitrogen*. HeLa cells were maintained at 37°C and 5% CO_2_ in Dulbecco's Modified Eagle Medium (DMEM) containing 10% fetal bovine serum, 100 U/ml penicillin, and 100 μg/ml streptomycin. For transient transfection, cells were plated on 6-well plates at a density of 4 × 10^5^ cells per well. Transfection by CMV-hEAAT_1_ and CMV-hEAAT_2_ plasmids was performed on the day following the plating in serum- and antibiotic-free DMEM using the FuGENE HD transfection reagent (Promega, USA) according to the manufacturer's instructions. Each well contained 2 μg DNA and 7 μl FuGENE HD. Cells were incubated for 4 h at 37°C and 5% CO_2_, then the medium was replaced with DMEM containing serum and antibiotics.

### Uptake assay in transfected HeLa cells

Aspartate uptake was investigated on the day following transfection using a previously described method (Velaz-Faircloth et al., 1996) with modifications. Untransfected HeLa cells were used as a control. All steps were performed in room temperature unless otherwise specified. Cells growing on 6-well plates were washed once with warm uptake buffer and then pre-incubated in 1 ml/well of uptake buffer for 15 min at 37°C and 5% CO_2._ The uptake buffer contained (in mM): 120 NaCl, 4.7 KCl, 1.2 KH_2_PO_4_, 2 CaCl_2_, 1.2 MgCl_2_, 10 D-glucose, 5 Tris-HCl, and 10 HEPES, and the pH was adjusted to 7.40 with NaOH. Subsequently, uptake was initiated by replacing the pre-incubation buffer with 1 ml/well of uptake buffer containing the substrate [20 μM D-aspartic acid (Sigma-Aldrich), 1% of which was D-[2,3-^3^H]-aspartic acid (PerkinElmer; 10.0 Ci/mmol)] either alone or in combination with 1 μM of BaCl_2_ (Sigma-Aldrich) or 2 μM of the EAAT_1/2_ inhibitor (3S)-[[3-[[4-(trifluoromethyl)benzoyl]amino]phenyl]methoxy]-L-aspartic acid (TFB-TBOA). Uptake was continued for 20 min at 37°C and 5% CO_2_ and then terminated by placing the cells on ice and washing rapidly three times with ice-cold 0.32 M sucrose. The cells were then immediately solubilized in 0.25 M NaOH (1 ml/well). Radioactivity of the solubilized samples was determined by scintillation counting using a Wallac WinSpectral 1414 liquid scintillation counter after addition of Optiphase HiSafe 3 scintillation cocktail (PerkinElmer). Instrument efficiency was 40%. Protein concentration in the samples was measured with the Bradford assay using the Sigma-Aldrich Bradford Reagent. The data are expressed in picomoles of substrate per milligram of protein.

### Centroid calculation

This method was previously proposed as a way to estimate the uptake efficiency: when the efficiency increases, the centroid of the transporter current becomes smaller because of the faster rise and decay times of the current (Diamond, 2005). Briefly, the centroid was calculated as the ratio of the first moment of the TC waveform to its area

$$TC\text{ centroid}=\frac{\int tf(t)dt}{\int f(t)dt}$$

The time frame was confined to the duration of TC waveform. Each data point was multiplied by its corresponding time point. The resulted waveform was integrated and divided by the integral of TC waveform.

### Statistical analysis

Averaged data are presented as mean ± standard error of mean (SEM). Statistical significance was assessed using, Student's *t*-test or One-Way ANOVA with *post-hoc* Tukey test, as indicated in the figure legends or in the text. A *P*-value of less than 0.05 was considered statistically significant.

## Results

**Figure 1:**
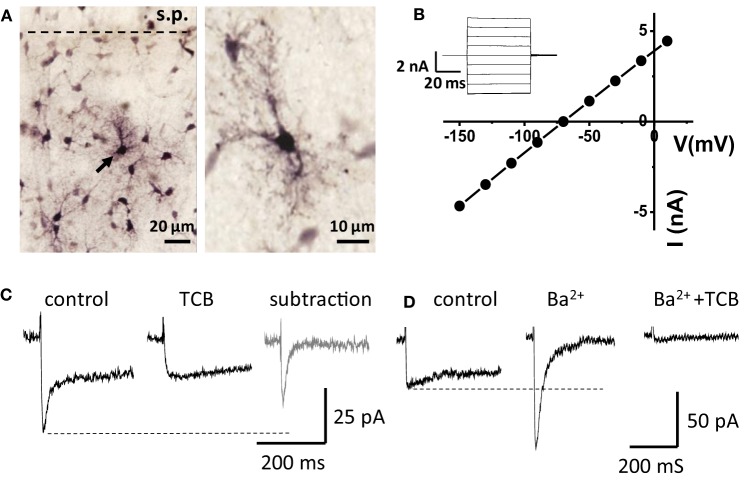
**Recordings from “passive” astrocytes reveal sensitivity of glutamate transporter current to Ba^2+^. (A)** Astrocytes in hippocampal CA1 *stratum radiatum* (stained with biocytin) s.p.—*stratum pyramidale*. Arrow indicates a patched astrocyte. **(B)** I-V relation of the patched astrocyte. *Inset*: membrane currents in response to voltage steps. **(C)** Astrocytic current induced by electrical stimulation of Schaffer collaterals in control and in the presence of glutamate transporter current blockers (TCB). *Gray trace*—“pure” transporter current obtained by subtracting the current in TCB from the control current. **(D)** Ba^2+^ (200 μM) abolishes the slow K^+^ component of the complex current and increases the amplitude of fast transporter current, which is consequently blocked by TCB (Ba^2+^ + TCB).

Whole-cell voltage-clamp recordings of transporter currents were performed in visually identified astrocytes in rat hippocampal slices. A subset of the recorded cells was filled with biocytin through the recording pipette, and *post-hoc* reconstruction of the cells revealed typical astrocytic morphology and staining of neighboring astrocytes due to biocytin diffusion through the gap-junctions (Figure 1A). Recorded cells had a relatively large hyperpolarized resting membrane potential (−81.4 ± 0.6 mV, *n* = 62, measured in current clamp mode), low membrane resistance (23 ± 2 MΩ, *n* = 62), and linear I-V relationship (Figure 1B).

**Figure 2:**
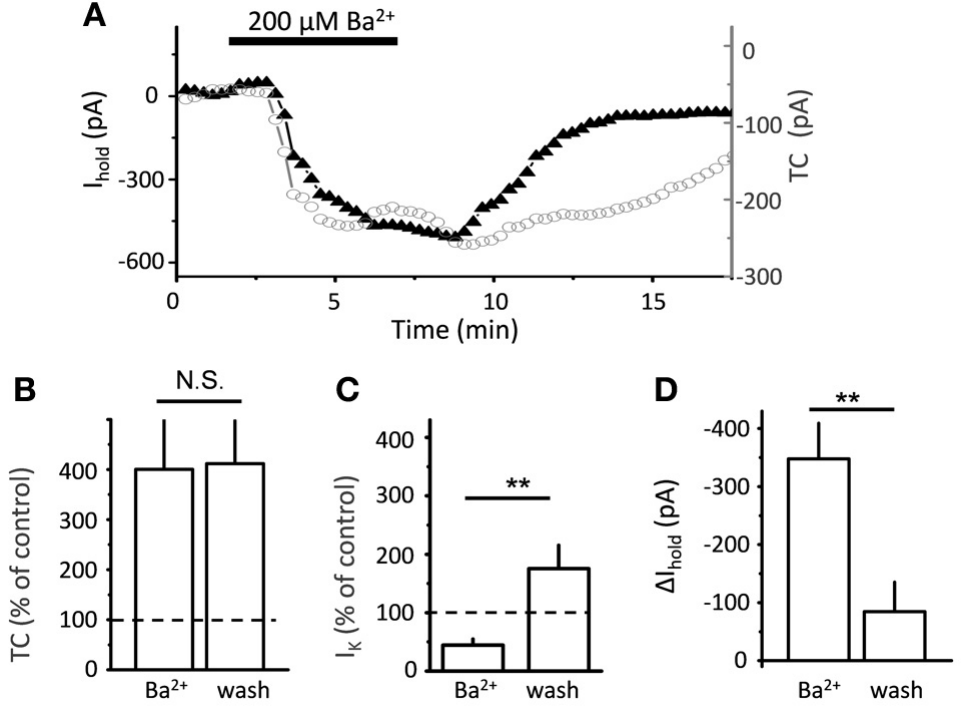
**Time-course of Ba^2+^ effect on transporter current and K^+^ current. (A)** Bath-applied 200 μ M BaCl_2_ produces downward shift in I_hold_ (black triangles), and increase in transporter current (TC, empty circles) in a single cell. **(B)** Summary graph of mean normalized transporter current (TC, *n* = 5) in the presence of Ba^2+^ and after 5 min of washout (wash). **(C)** Summary graph of normalized K^+^ current (*n* = 5) in the presence of Ba^2+^ and after 10 min of washout (wash) **(D)** Summary graph of mean Δ I_hold_ (*n* = 5) in the presence of Ba^2+^ and after 10 min of washout (wash). Error bars—SEM; ^**^P < 0.01; N.S.—non significant; One-Way ANOVA *post-hoc* Tukey test.

Electrical stimulation of the Schaffer collaterals elicited a biphasic inward current with fast and slow components in astrocytes voltage-clamped at −80 mV. Glutamate transporters mediated the fast current component, because the current was fully blocked by a mixture of transporter blockers comprising 100 μM TBOA, 50 μM dihydrokainate, and 100 μM threo-hydroxy-aspartate (Figure 1C). We assumed that the slow current component was mediated by K_ir_ (Kofuji and Newman, 2004) and would be sensitive to Ba^2+^. Therefore, to isolate the glutamate transporter current, Ba^2+^ was added to the bath at the same concentration (200 μM) as previously described (D'Ambrosio et al., 2002; De Saint Jan and Westbrook, 2005). Along with the expected K^+^ current blockade, Ba^2+^ strongly increased the amplitude of the transporter current (thereafter also referred to as “potentiation of the transporter current”; Figure 1D). A similar increase in transporter current was previously reported in response to a cocktail of K^+^ channel blockers (including Ba^2+^) and attributed to decrease in membrane conductance (Ge and Duan, 2007). Indeed, K_ir_ blockade increased input resistance (R_i_, 24 ± 2 MΩ in control and 31 ± 3 MΩ in Ba^2+^, *n* = 16, *P* = 0.002 for difference, paired *t*-test), potentiation of the transporter current could be explained by increase in the membrane length constant, which is proportional to the square root of R_i_. In this case, a higher membrane time constant would lead to a larger contribution of transporter currents originating in distant astrocytic processes into the current recorded in the soma, and the time-courses of the effects of Ba^2+^ on transporter current and on membrane conductance would correlate. We found, however, that augmentation of transporter current started earlier than the change in K^+^ current and in holding current at the beginning of Ba^2+^ application (Figure 2). Moreover, the transporter current augmentation persisted longer upon Ba^2+^ washout (transporter current in BaCl_2_: 400 ± 99% of control, *n* = 5; 5 min washout: 411 ± 86% of control, *n* = 5, One-Way ANOVA, *F*_(2, 12)_ = 5.42, *P* = 0.021; *post-hoc* Tukey test, *P* = 0.587 for difference between BaCl_2_ and washout; Figure 2B) than did suppression of the K^+^ current (K^+^ current in BaCl_2_: 44 ± 10% of control, *n* = 5; 5 min washout: 175 ± 40% of control, *n* = 5, One-Way ANOVA, *F*_(2, 12)_ = 10.71, *P* = 0.002; *post-hoc* Tukey test, *P* = 0.002 for difference between BaCl_2_ and washout; Figure 2C) and holding current (Δ I_hold_ in BaCl_2_: 347 ± 61 pA, *n* = 5; and 84 ± 51 pA at 5 min of washout, *n* = 5, One-Way ANOVA, *F*_(2, 12)_ = 23.83, *P* < 0.001; *post-hoc* Tukey test, *P* < 0.001 for difference between BaCl_2_ and washout; Figure 2D), suggesting that the change in the membrane conductance did not contribute to the effect of Ba^2+^ on the transporter current.

**Figure 3:**
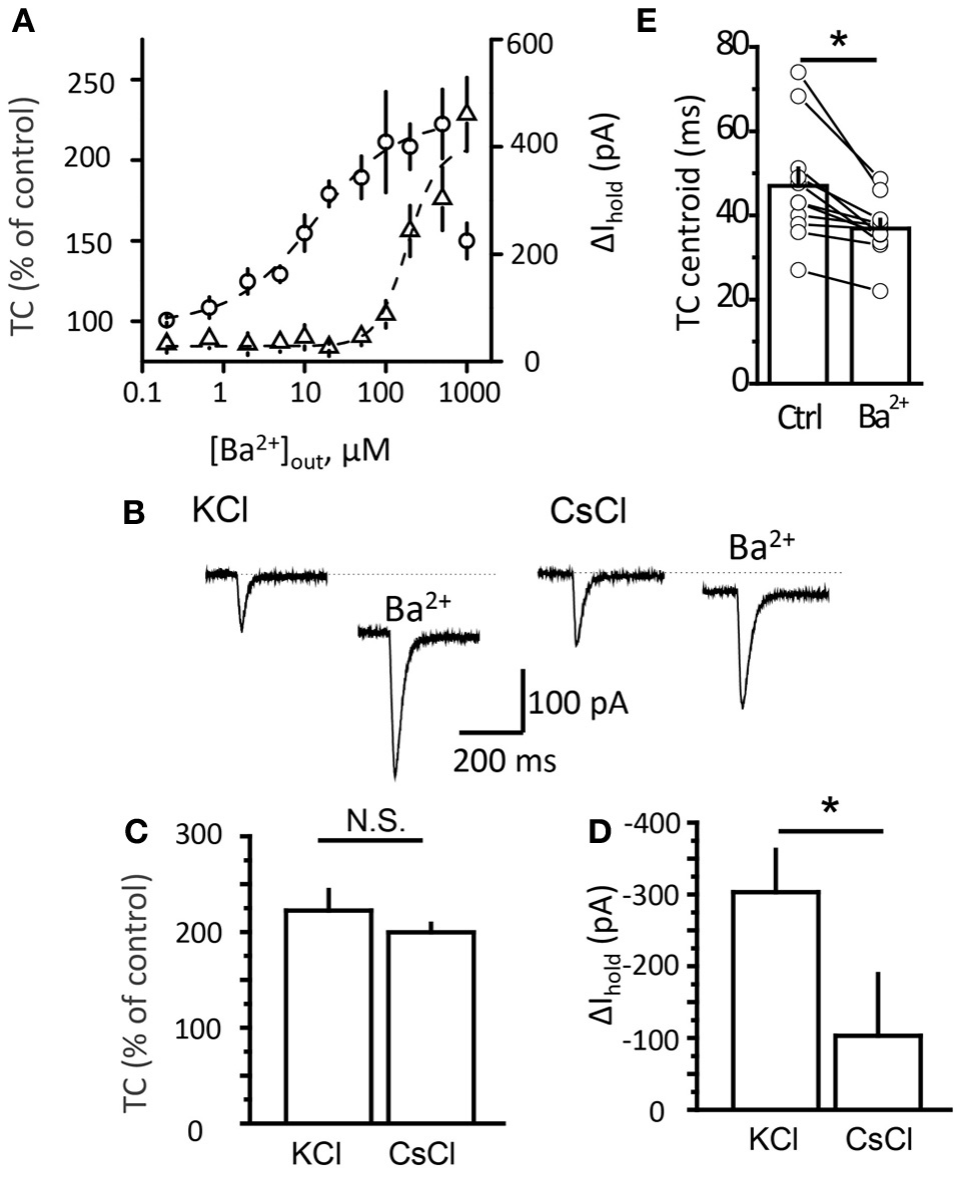
**Potentiation of transporter current is independent of K_ir_ blockade. (A)** Dose-response curve of TC (circles) and Δ I_hold_ (triangles) changes. **(B)** Transporter current (TC) elicited by local glutamate uncaging in control and in 200 μM Ba^2+^. The downward shift in the holding current was less pronounced in Cs^+^-loaded cells (CsCl). The TC amplitude increased to a similar degree in both cases. **(C,D)** Mean Ba^2+^ effect on transporter current (TC, *B*) and I_hold_ (Δ I_hold_, *C*) for CsCl- and KCl-based intracellular solutions. **(E)** Transporter current centroids in control and in 20 μM Ba^2+^. Error bars—SEM. ^*^P < 0.05; N.S.—non significant; paired **(E)** and unpaired **(C,D**) *t*-test.

Because the Ba^2+^ concentration gradually increases in the tissue after adding the drug to the perfusion system, the faster effect on transporter current suggests its higher sensitivity to Ba^2+^ than K^+^ current. One possibility is that low Ba^2+^ concentrations increased presynaptic glutamate release, leading to a larger transporter current. To test this we replaced synaptic stimulation with local glutamate uncaging. Because no action potential or synaptic activation was involved, a K^+^ current did not accompany the transporter current in this case. We plotted dose-response curves for the effect of Ba^2+^ on the transporter current and Δ I_hold_ (Figure 3A). Consistent with the synaptic stimulation results, the half-maximal effective concentration (EC_50_) for the transporter current was 13 μM, while EC_50_ for Δ I_hold_ was 183 μM. Strikingly, low micromolar Ba^2+^ concentrations (1-50 μM) effectively increased the transporter current, but had no detectable effect on the holding current. This finding suggests that the Ba^2+^-induced potentiation of transporter current was not mediated by its effect on presynaptic glutamate release.

Although the EC_50_ for the transporter current and for the holding current differed, there is a possibility that Ba^2+^ affected K^+^ conductance in distal processes which cannot be detected with somatic recordings. Activation of the transporters during synaptic stimulation may indeed occur at such distal astrocytic processes. This possibility can be ruled out by the result of uncaging experiments, because the glutamate uncaging spot was located at the soma, right at the place where the holding current and transporter current were occurred in this case. We then further tested the contribution of the membrane conductance change to the effect of Ba^2+^ on the transporter current by blocking K^+^ channels from inside the cell with Cs^+^ (Janigro et al., 1997; Tang et al., 2009). Although Cs^+^ blocks K^+^ channels, it can substitute for K^+^ at the counter-transport site of glutamate transporters (Barbour et al., 1991). We compared the effect of Ba^2+^ on the holding current and transporter current induced by local glutamate uncaging in astrocytes recorded with K^+^-based and Cs^+^-based intracellular solutions (Figure 3B). Ba^2+^ at 500 μM potentiated the transporter current in Cs ^+^-loaded astrocytes to the same extent as in astrocytes recorded with the K^+^-based intrapipette solution (transporter current increased to 223 ± 21% of control with Cs^+^ solution, *n* = 6; and to 200 ± 9% of control with K^+^ solution, *n* = 5; *P* = 0.66, unpaired *t*-test; Figure 3C). In contrast, the effects of Ba^2+^ on the holding current were significantly lower in cells recorded with Cs^+^ (change in holding current, Δ I_hold_: −103 ± 86 pA, *n* = 6, with Cs^+^ solution; and −303 ± 59 pA, *n* = 5 with K^+^ solution; *P* = 0.02, unpaired *t*-test; Figure 3D).

**Figure 4:**
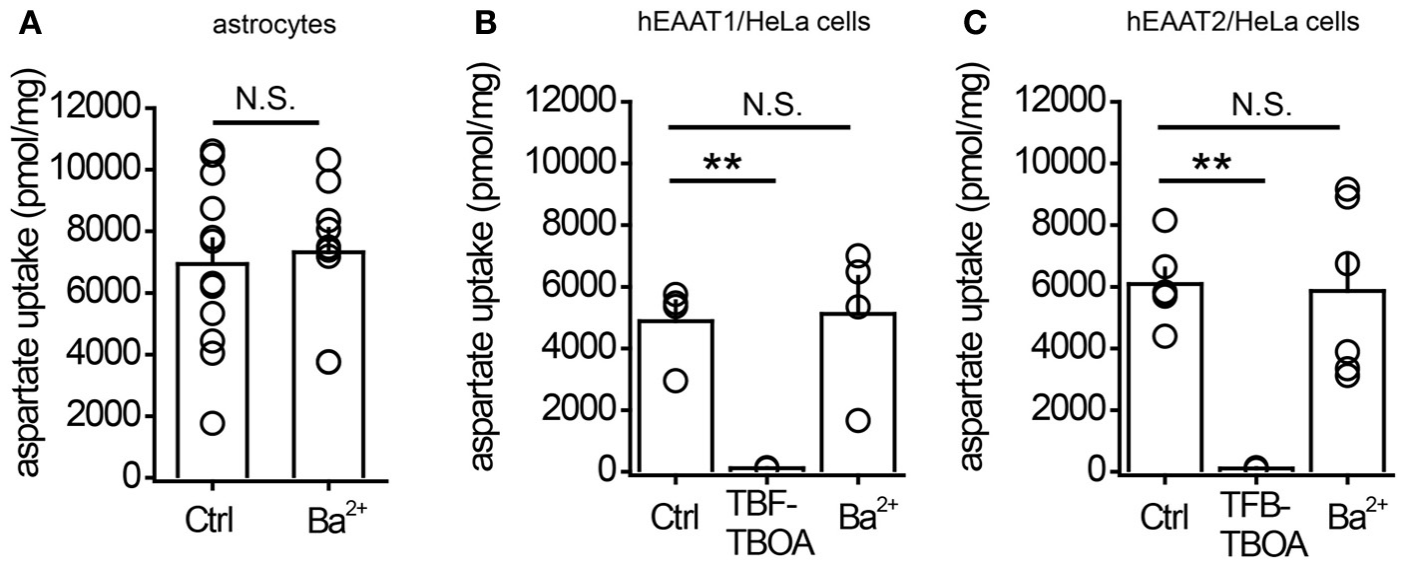
**1μM Ba^2+^ does not potentiate aspartate uptake by cultured astrocytes or by HeLa cells expressing glutamate transporters. (A)** Summary graph of aspartate uptake by cultured astrocytes in control conditions (Ctrl) or in the presence of 1 μM BaCl_2_ (Ba^2+^). **(B)** Summary graph of aspartate uptake by HeLa cells expressing EAAT1 in control conditions (Ctrl), in the presence of 2 μM TFB-TBOA, or in the presence of 1 μM BaCl_2_ (Ba^2+^). **(C)** Summary graph of aspartate uptake by HeLa cells expressing EAAT2 in control conditions (Ctrl), in the presence of 2 μM TFB-TBOA, or in the presence of 1 μM BaCl_2_ (Ba^2+^). Error bars—SEM. ^**^P < 0.01; N.S.—non significant; unpaired *t*-test.

Next we measured the centroid of the transporter current under baseline conditions and in the presence of different concentrations of Ba^2+^ (Figure 3E). This method was previously proposed as a way to estimate the uptake efficiency: when the efficiency increases, the centroid of the transporter current becomes smaller because of the faster rise and decay times of the current (Diamond, 2005). Here, we also detected a Ba^2+^-mediated decrease in the centroid of the uncaging-induced transporter current (from 47 ± 4 ms at control to 37 ± 2 ms in 20 μM Ba^2+^, *n* = 11, *P* = 0.002 paired *t*-test). This suggests that potentiation of transporter current may be associated with potentiation of glutamate uptake. To test this directly we studied the Ba^2+^ effect on uptake efficiency in cultured astrocytes. However, 1 μM Ba^2+^ did not significantly increase aspartate uptake (control: 6938 ± 797 pmol/mg, *n* = 12; Ba^2+^: 7324 ± 758 pmol/mg, *n* = 9; *P* = 0.37, unpaired *t*-test; Figure 4A). In cells that were transfected with the CMV-hEAAT_1_ or CMV–hEAAT_2_ plasmids, the aspartate uptake was practically abolished by 2 μM TFB-TBOA in the case of both transporters EAAT1 and EAAT2 expressed in HeLa cells (heterologous system, Figures 4B,C). The aspartate uptake was almost fully suppressed by 2 μM TFB-TBOA in case of both transporters (EAAT1 control uptake efficiency: 4881 ± 647 pmol/mg, *n* = 6; vs. TFB-TBOA: 115 ± 8 pmol/mg, *n* = 4; *P* < 0.001, unpaired *t*-test; EAAT2 control uptake efficiency: 6085 ± 508 pmol/mg, *n* = 4; vs. TFB-TBOA: 107 ± 13 pmol/mg, *n* = 4; *P* < 0.001, unpaired *t*-test), suggesting that the transporters were indeed expressed in these cells. Cells that were not transfected retained only a very small amount of aspartate, comparable to transfected cells treated with TFB-TBOA (data not shown). Consistent with the astrocyte results the uptake was not significantly affected by 1 μM Ba^2+^ in either type of HeLa cell (EAAT1 Ba^2+^: 5120 ± 1205 pmol/mg; *P* = 0.86 for difference with the control, unpaired *t*-test; EAAT2 Ba^2+^: 5862 ± 1137 pmol/mg; *P* = 0.86 for difference with the control, unpaired *t*-test). These findings suggest that although Ba^2+^ increases the amplitude of the glutamate transporter currents and changes their kinetics at concentration which do not affect K_ir_, it does not potentiates glutamate uptake.

## Discussion

The main finding of the present study is the potentiating effect of low micromolar Ba^2+^ on astrocytic transporter current which is not associated with increased glutamate uptake. These Ba^2+^ concentrations do not change membrane conductance which could explain the potentiation. Moreover Ba^2+^ decreases the transporter current centroid which was used to estimate the efficiency of glutamate uptake (Diamond, 2005; Scimemi et al., 2009; Thomas et al., 2011). This discrepancy suggests that the difference in transporter current kinetics may not be due to differences in glutamate uptake. Therefore additional evidence such as activation of extrasynaptic receptors (e.g., NMDA receptors) is required to supplement the transporter current measurements (Scimemi et al., 2009; Thomas et al., 2011; Tanaka et al., 2013).

Although we find that Ba^2+^ potentiates the transporter current in hippocampal astrocyte the molecular mechanism of this action is unclear. Indeed, blockade of K_ir_ with Ba^2+^ produced a downward shift in the holding current recorded in hippocampal astrocytes, which is consistent with membrane depolarization. In principle, depolarization in poorly voltage-clamped astrocytic processes should reduce the efficiency of electrogenic glutamate transporters. To the contrary, we detected a significant increase in glutamate transporter currents induced either by synaptic stimulation or by local glutamate uncaging. Potentiation of uncaging-mediated transporter current suggests that this phenomenon is independent of the effects of Ba^2+^ on presynaptic release probability. However, we cannot exclude that a part of potentiation in the case of synaptically released glutamate can be explained by presynaptic effects of Ba^2+^, which were not tested in this study. Importantly, a significant effect on transporter current was observed at low micromolar Ba^2+^ concentrations that produced no measurable effect on the holding current. Thus, Ba^2+^ increased the amplitude of glutamate transporter current independently of K_ir_ blockade. It is possible that Ba^2+^ modulated transporter currents uncoupled from glutamate uptake (Bergles et al., 2002).

Although our data suggest that Ba^2+^ acts to increase the amplitude of glutamate transporter current, the mechanism for its action remains unclear. Here we tested the effect of Ba^2+^ on synaptically induced transporter currents and transporter currents mediated by glutamate uncaging. Although glutamate uncaging is a useful method it cannot entirely mimic synaptically released glutamate. For example, to achieve measurable transporter current from single uncaging spot we located the spot at the soma and used 10 ms uncaging duration. This is in contrast to synaptic glutamate release that occurs not only at synapses close to the soma but also at synapses close to the distal astrocytic processes and lasts <1 ms at each synapse. Moreover, recent reports suggest existence of Ca^2+^ microdomains associated with fragments of astrocytic processes seen in a single imaging plane that are quite distinct from somatic Ca^2+^ signaling (Shigetomi et al., 2013; Tong et al., 2013). It is also likely that properties of glutamate uptake differ between astrocytic soma and processes. Thus, future studies are needed to identify the mechanism by which Ba^2+^ exerts its action on transporter current. An understanding of the mechanism might have important implications for both research and the development of neuroactive drugs.

## Authors' contribution

Ramil Afzalov, Evgeny Pryazhnikov, and Pei-Yu Shih performed slice experiments, analyzed the data and prepared the figures; Elena Kondratskaya performed preliminary experiments and discovered the potentiating effect of barium, Svetlana Zobova prepared astrocyte cultures, Sakari Leino and Outi Salminen preformed uptake assays, Ramil Afzalov, Leonard Khiroug, and Alexey Semyanov planned the study and wrote the manuscript.

### Conflict of interest statement

The authors declare that the research was conducted in the absence of any commercial or financial relationships that could be construed as a potential conflict of interest.
